# Supplementary material for: Investigating the influence of physiologically relevant hydrostatic pressure on CHO cell batch culture
Source: Sci Rep. 2021 Jan 8;11:162. doi: 10.1038/s41598-020-80576-8 (PMC7794228; doi:10.1038/s41598-020-80576-8)
Supplement: Supplementary file 1 — Supplementary Information 1. [file 41598_2020_80576_MOESM1_ESM.zip › S1/IgG/Feb14 0 30 60 90/60mmHg Feb3.pdf]

Data File C:\Users\P...emStation\1\Data\TK_CHO_IgG 2019-02-14 10-52-34\018-10-ML 60 Feb3.D
Sample Name: ML 60 Feb3

===

Acq. Operator : SYSTEM Seq. Line : 18
Sample Operator : SYSTEM
Acq. Instrument : HPLC-0039 Location : 10
Injection Date : 2/14/2019 12:07:35 PM Inj : 1
Inj Volume : 100.000 µl
Method : C:\Users\Public\Documents\ChemStation\1\Data\TK_CHO_IgG 2019-02-14 10-52-34 \CHO_IgG_LC.M (Sequence Method)
Last changed : 2/8/2019 3:17:23 PM by SYSTEM

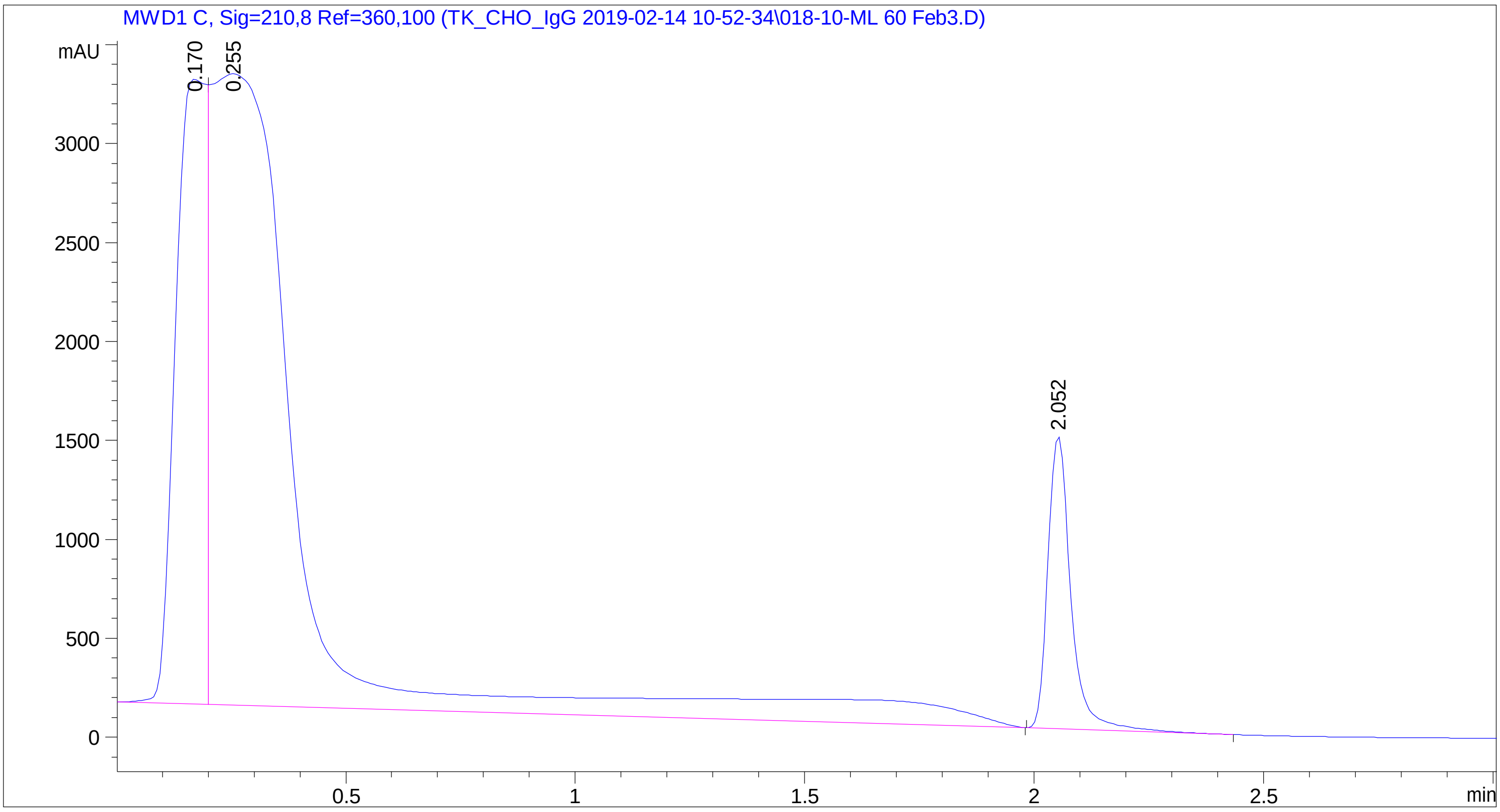

===

Area Percent Report

===

Sorted By : Signal
Multiplier : 1.0000
Dilution : 1.0000
Use Multiplier & Dilution Factor with ISTDs

Signal 1: MWD1 C, Sig=210,8 Ref=360,100

Peak #	RetTime [min]	Type	Width [min]	Area [mAU*s]	Height [mAU]	Area %
1	0.170	BV	0.0702	1.46823e4	3158.50244	23.5486
2	0.255	VB	0.1954	4.24915e4	3190.24756	68.1512
3	2.052	BBA	0.0528	5175.06152	1480.58093	8.3002

Totals : 6.23489e4 7829.33093

===

*** End of Report ***
